# A newly developed cloning technique in sturgeons; an important step towards recovering endangered species

**DOI:** 10.1038/s41598-019-46892-4

**Published:** 2019-07-18

**Authors:** Effrosyni Fatira, Miloš Havelka, Catherine Labbé, Alexandra Depincé, Martin Pšenička, Taiju Saito

**Affiliations:** 1 Faculty of Fisheries and Protection of Waters, South Bohemian Research Center of Aquaculture and Biodiversity of Hydrocenoses, University of South Bohemia in České Budějovice, Zátiší 728/II, 389 25 Vodňany, Czech Republic; 2 South Ehime Fisheries Research Center, Ehime University, Ainan, Ehime 798-4206 Japan; 3 INRA, Fish Physiology and Genomics department, Campus de Beaulieu, F-35000 Rennes, France

**Keywords:** Cloning, Embryology

## Abstract

Several steps of sturgeon somatic cell nuclear transfer (SCNT) have been recently established, but improvements are needed to make it a feasible tool to preserve the natural populations of this group of endangered species. The donor cell position inside the recipient egg seems to be crucial for its reprogramming; therefore by injecting multiple donor somatic cells instead of a single cell with a single manipulation, we increased the potential for embryo development. Using the Russian sturgeon *Acipenser gueldenstaedtii* as a multiple cell donor and sterlet *Acipenser ruthenus* as the non-enucleated egg recipient, we obtained higher proportion of eggs developing into embryos than previously reported with single-SCNT. Molecular data showed the production of a specimen (0.8%) contained only the donor genome with no contribution from the recipient, while two specimens (1.6%) showed both recipient and donor genome. These findings are the first report of donor DNA integration into a sturgeon embryo after interspecific cloning. In all, we provide evidence that cloning with the multiple donor somatic cells can be feasible in the future. Despite the fact that the sturgeon cloning faces limitations, to date it is the most promising technique for their preservation.

## Introduction

The Acipenseridae is an ancient family that faces internal and external threats including the loss of species genetic integrity through frequent interspecific hybridization^1^, habitat degradation, and overfishing for their roe processed into caviar^2^. Dramatic decrease in sturgeon populations attracted attention of the International Union for Conservation of Nature (IUCN) that categorized them as the most critically endangered, more than any other group of species. Indeed, all 27 sturgeon species are on the IUCN Red List of threaten species with 17 categorized as critically endangered and four considered to be extinct^3^.

Somatic cell nuclear transfer (SCNT) is an animal cloning technique that can aid conservation of species on the verge of extinction^4–11^. A single somatic cell from an endangered species can be reprogrammed to totipotency when placed in a favorable cytoplasmic environment of an easily reared species, producing an organism containing exclusively donor genomic material. Despite the demonstrated advantages of the method, SCNT is a challenging multi-step technique with low success even in model fish species^12–17^. The first crucial steps in sturgeon SCNT have been recently established and resulted in early embryogenesis of the nuclear transplants (NTs)^11^. However, utilizing a single fin cell harvested from an albino sterlet, *Acipenser ruthenus* and Russian sturgeon, *Acipenser gueldenstaedtii* transplanted into non-enucleated eggs from sterlet achieved low development rate of reconstructed NTs, of 18.1% and 12%, respectively^11^.

The current study aims to improve the sturgeon SCNT technique by increasing the number of developing NTs that would be surrogate for gamete production. Introduction the donor genome into the recipient egg, with the long-term goal that the germline will produce the desired gametes. Results of studies in goldfish *Carassius auratus* have suggested that the cell injection position and depth inside the recipient egg are critical for donor cell reprogramming^18^. A single fin cell could be easily found in a non-favorable position inside the larger recipient egg. Egg size varies in sturgeon species. Eggs of sterlet are smaller than those of Russian sturgeon and beluga, *Huso huso* (diameter 1.8–2.8 mm, 2.8–3.8 mm and 3.3–4.5 mm, respectively)^19^. Although sterlet eggs are the smallest among sturgeons^19^, they are considerably larger than a single fin cell (~8 μm). No technique is currently available that will place the donor cell in the specific area of the recipient egg most conducive to its reprogramming. Therefore, SCNT is basically a blind technique, particularly challenging when using large recipient eggs as in sturgeon.

In the present study we injected multiple donor somatic cells instead of a single cell by a single manipulation, conducting multiple somatic cell nuclear transfer (mSCNT). The newly developed cloning technique increases the potential for the donor fin cell to be placed in the most favorable position inside the recipient sterlet egg and reprogrammed. We used sturgeon species most commonly exploited for caviar, the Russian sturgeon and the beluga^19^, as donor species. Females and males Russian sturgeon are reproductively mature at 10–16 and 8–13 years, respectively^20^, and beluga at 15–18 and 10–15 years, respectively^21^. These species are categorized as critically endangered^3^, and therefore, fin tissue is an excellent source of donor genomic material, as the harvesting does not cause irreparable damage to the fish^10,22^. As a recipient cytoplasmic environment, non-enucleated and non-activated eggs of sterlet provide clear benefits^11^. Sterlet, classified as a vulnerable species^3^, displays characteristics that make it a model species of the sturgeon family. It begins reproducing much earlier than the two mentioned species, 5–8 years in females and at 3–5 years in males^23^. Sterlet females spawn every 1–2 years^23^ while Russian sturgeon and beluga spawn at 4–6^20^ and 3–4 years^21^ intervals, respectively. The use of sterlet as a recipient species and beluga as donor species has benefits for molecular genotyping using recently developed species-specific primers allowing routine identification of the NTs origin^24^.

The current study improves the state of the art of interspecific SCNT for multiplying endangered animals. We transplanted multiple fin cells of albino sterlet, Russian sturgeon, or beluga into non-enucleated and non-activated sterlet eggs. We compared two numbers of fin cells in their ability to trigger embryonic development of the multiple nuclear transplants (mNTs). We assessed the donor cell fate in the developing mNTs and monitored embryo development. Finally, we assessed the donor cell genomic contribution to the developing transplants using molecular markers. The results of SCNT reported here represent a baseline that will contribute to progress in cloning Russian sturgeon and beluga for conservation purposes. Most importantly, the current research provides evidence that large critically endangered rare animals can be generated via the powerful interspecific SCNT technique.

## Results

### Importance of shallow donor cell injection in the animal pole of recipient egg

To evaluate the effect of deep donor cell injection in the central region of the recipient egg, we monitored development resulting from intraspecific and interspecific single-SCNT. No, initial cleavage was shown in the transplants and therefore shallow injection in the animal pole of the recipient egg was performed in the subsequent experiments.

### Effect of the quantity of transferred donor cells in mNTs development

**Figure 1:**
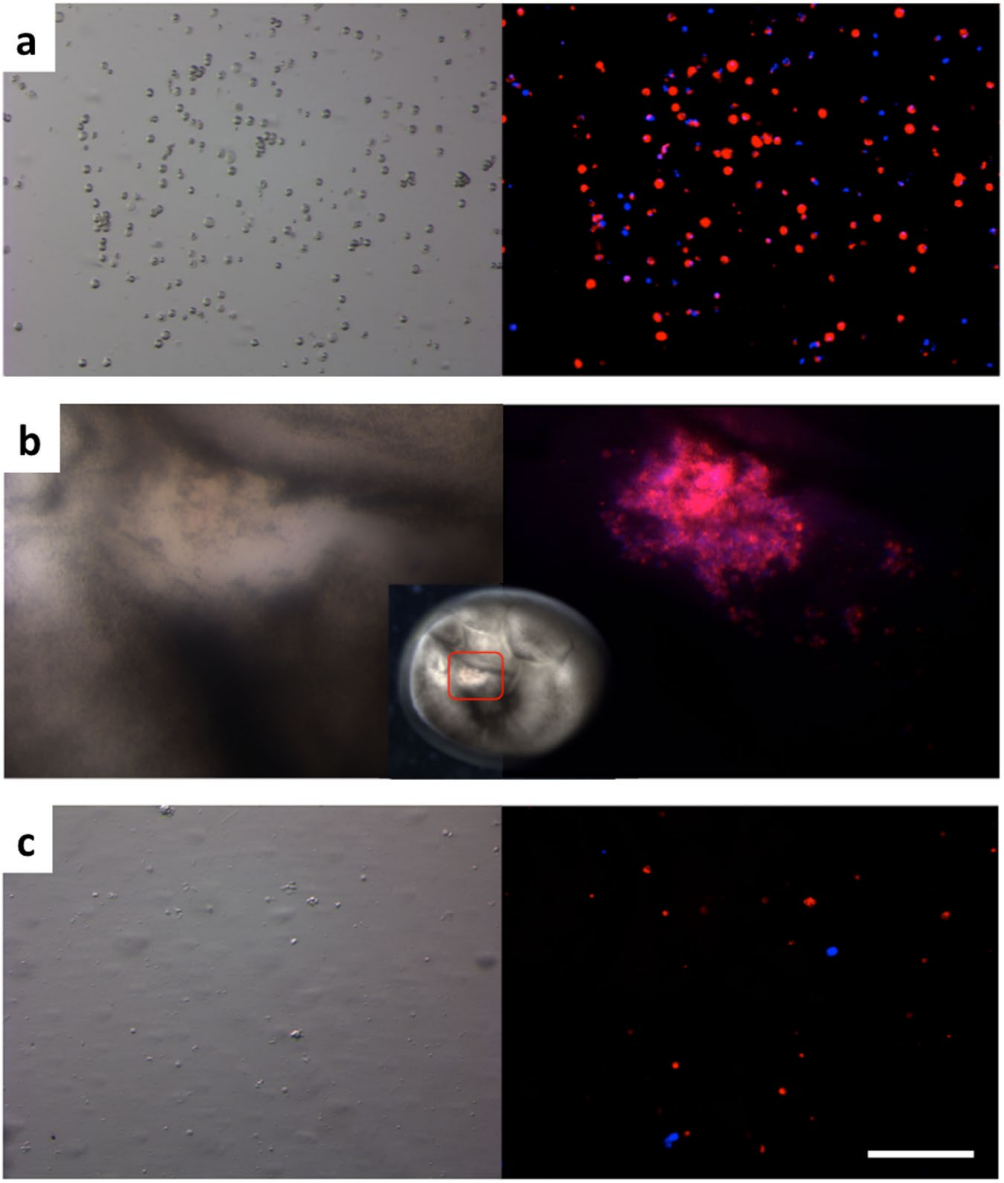
*In vivo* fluorescence analysis of fresh dissociated fin cells from Russian sturgeon. The left column represents bright field images while the right column shows the merging of UV and DsRED images. (**a**) Freshly dissociated fin cells before transplantation. The red color indicates the fin cell plasma membrane while the blue color indicates the released fin cell nuclei in cells with altered or striped off plasma membranes. (**b**) Visualization of the multiple fin cells inside the 8-cell stage developing Russian sturgeon mNT (corresponding to 6 hpa). The red color indicates the fin cell plasma membrane (intact or striped off) and the violet color indicates existence of released nuclei (blue color) that overlapped with the fin cell plasma membrane (red color). (**c**) Nuclei appeared after smashing the 8-cell stage Russian sturgeon mNT. Scale bar corresponds to 200 μm.

To evaluate mNT embryo development with respect to the number of transplanted fin cells, we monitored development resulting from two quantities of fin cells from Russian sturgeon (~330 ± 12 and ~55 ± 3/egg) and from beluga (~240 ± 18 and ~40 ± 2/egg) transplanted into sterlet eggs (Table 1). A significantly greater number of developing mNTs was observed with the higher number of injected cells compared to the lower quantity: 4-fold in Russian sturgeon mNTs (*P* < 0.001) and 1.6-fold in beluga mNTs, (*P* < 0.05) (Supplementary Fig. 1). The rate of mNTs development decreased in successive stages, with the number of both Russian sturgeon and beluga mNTs reaching gastrula lower compared to blastula (Table 1, Supplemetary Fig. 1). Considering these results, in subsequent experiments, we used the higher tested number of fin cells for mSCNT.

**Table 1:** Comparison of mNTs developmental rate after different number of fin cells from Russian sturgeon or beluga were injected into sterlet eggs.

Fin-cells donor species	Fin-cells/egg	Number of injected eggs	Blastula (%)	Gastrula-Blastopore formation (%)
Russian sturgeon	~330 ± 12	22	13 (59.1)	1 (4.5)
	~55 ± 3	34	5 (14.7)	0 (0)
Beluga	~240 ± 18	74	38 (51.4)	4 (5.4)
	~40 ± 2	56	18 (32.1)	1 (1.8)

### The fate of fin cells before and after microinjection

We visualized *in vivo* the fate of the fin cells immediately after dissociation as well as within the developing mNT. Two dyes were visible after the freshly dissociated fin cells from Russian sturgeon: the cells with intact or altered plasma membranes (red stain hiding the nucleus staining), as well as the released nuclei without plasma membrane staining (blue stain) (Fig. 1a). Inside the 8-cell stage developing Russian sturgeon mNT at six hours post-activation (hpa), due to many donor fin cells injected into a single area inside the egg, the released nuclei (blue stain) and the fin cell plasma membrane (red stain) are overlapping, resulting in a violet color. The violet color indicates the existence of blue stained released nuclei that overlap with red stained fin cell plasma membranes (intact or striped off) (Fig. 1b). We cannot exclude the possibility that some fin cell plasma membranes inside the sterlet egg collapsed, increasing the number of blue cells observed. After smashing the 8-cell stage Russian sturgeon mNT, both nuclei and plasma membranes were observed, indicating that plasma membranes of donor cells are not digested after injecting into the cytoplasm of oocyte, unlike the previous result reported in goldfish cloning^18^ (Fig. 1c). The materials that appeared only in a bright filter image are probably debris from the smashed mNT (Fig. 1c).

### Histology

**Figure 2:**
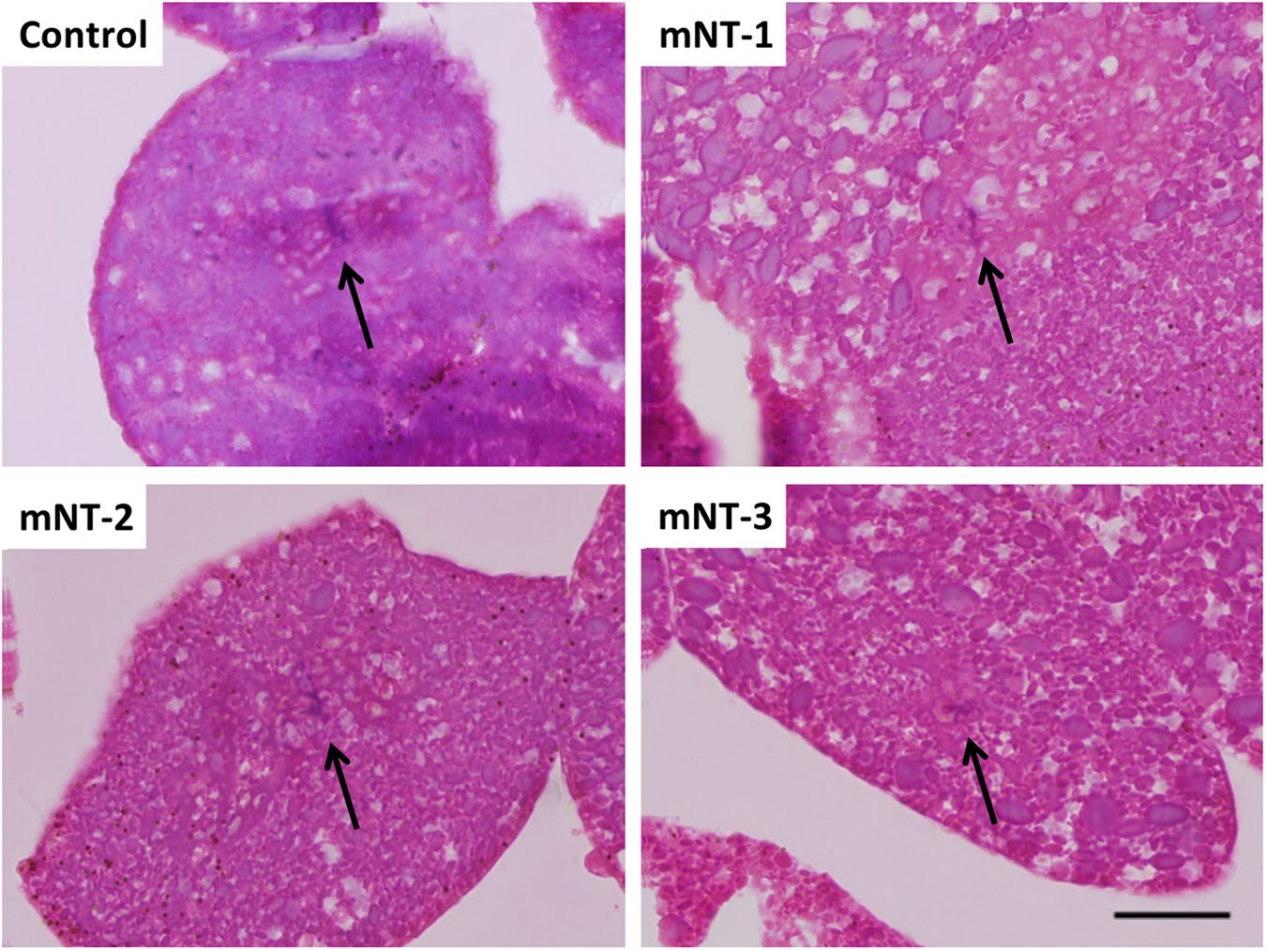
Histological sections of early blastula stage (256-cell) sterlet embryo (Control) and three Russian sturgeon mNTs (mNT-1, mNT-2 and mNT-3) at the same age. Black arrows indicate the location of the nuclei inside single blastomeres. Scale bar corresponds to 25 μm.

The presence of a nucleus inside a single blastomere of early blastula Russian sturgeon mNTs (256-cell stage, corresponding to 11 hpa) and sterlet control at the same time post-activation showed that the formed blastomeres were the result of embryological cleavage and not a mechanical stress response to microinjection (Fig. 2).

### Effectiveness of mSCNT technique and mNTs development

The albino sterlet mNTs that displayed early cleavage reached blastula (44.4%) and two embryos (11.1%) succesfully formed the blastopore in gastrula before ceasing development. After the Russian sturgeon mSCNT, 85 Russian sturgeon mNTs that displayed early cleavage reached blastula (66.4%), and seven formed the blastopore in the gastrula (5.5%) but did not develop further. Sixty-eight beluga mNTs that displayed initial cleavage reached blastula (52.3%), eight formed the blastopore (6.2%), and three (2.3%) reached 2/3 of epiboly in the gastrula. In conclusion, all mNTs that displayed initial cleavage fullfilled blastula and either ceased development or developed further. After *in vitro* fertilization, more than 90% of sterlet control embryos showed development (n = 320). No extender-solution-only injected negative control (n = 100), showed cleavage furrows, as no somatic cell was injected (Table 2).

**Table 2:** Developmental rates of the reconstructed embryos (albino sterlet mNTs or Russian sturgeon mNTs or beluga mNTs) after transplantation of fin-cells originated from albino sterlet or Russian sturgeon or beluga, respectively, into sterlet eggs (~329 ± 2 or ~330 ± 12 or ~240 ± 18 fin-cells/egg, respectively).

Experimental group	Total number of eggs	Blastula (%)	Gastrula (%)	
			Blastopore formation	2/3 Epiboly
Fertilized control	351	320 (91.2)	310 (88.3)	310 (88.3)
Extender-injected control	100	0 (0)	0 (0)	0 (0)
Albino sterlet mNTs	18	8 (44.4)	2 (11.1)	0 (0)
Russian sturgeon mNTs	128	85 (66.4)	5 (3.9)	0 (0)
Beluga mNTs	130	68 (52.3)	8 (6.2)	3 (2.3)

Sterlet control groups are presented at each developmental stage respectively to mNTs.

### Phenotype of resulted mNTs

**Figure 3:**
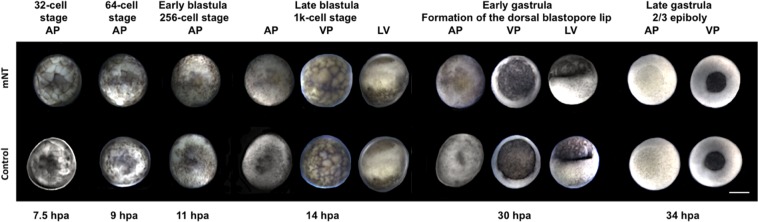
Comparison of the embryonic development between a beluga mNT and a sterlet control embryo. The embryonic stages, from early developmet (32-cell stage) until late gastrula (2/3 epiboly) are presented with the respective hours post activation (hpa) of the eggs. Animal pole (AP), vegetal pole (VP), lateral view (LV), scale bar corresponds to 1 mm.

All mNTs produced developed normally, following the pattern of the sterlet control group. Immediately prior to cessation of development, the majority of mNTs formed the dorsal blastopore lip in the gastrula, with the exception of three beluga mNTs that developed further, forming 2/3 epiboly of the gastrula (Fig. 3).

**Figure 4:**
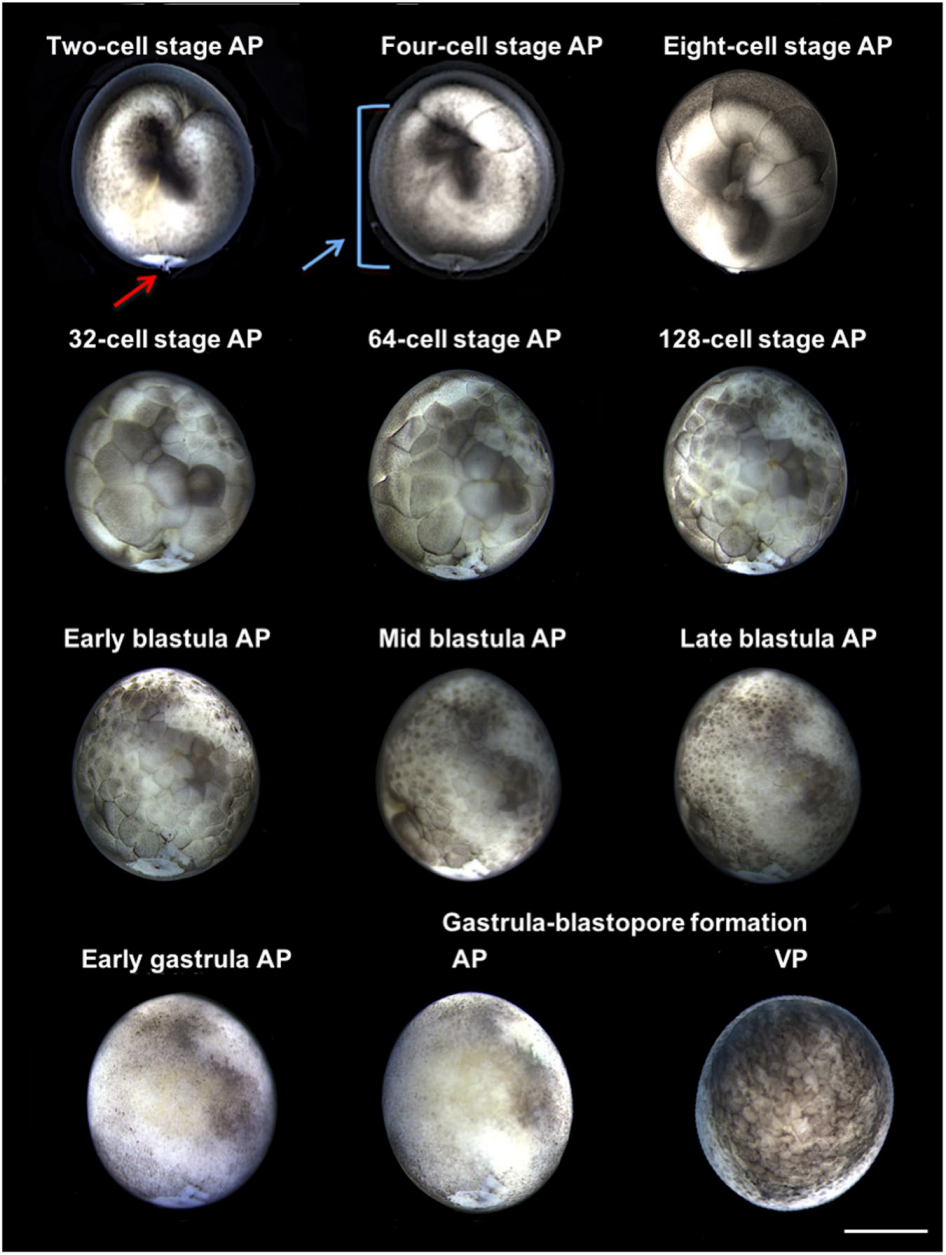
Development of a Russian sturgeon mNT that exhibit large blastomeres together with the normal size ones. The red arrow shows an injury position due to microinjection that remains throughout the development (gastrula-blastopore formation). The blue arrow is showing the area where the large blastomeres were created, mostly on the center and left side of the mNT. These blastomeres formed at the 4-cell developmental stage and were no longer visible in the late blastula stage. Animal pole (AP), vegetal pole (VP), scale bar corresponds to 1 mm.

Two developing Russian sturgeon mNTs (2.4%) formed blastomeres with atypically large-volume cells at the 4-cell stage, corresponding to 5 hpa. However, at the late blastula corresponding to 14 hpa, they exhibited no phenotypic difference from the sterlet control group; both developed the blastopore in the gastrula and then ceased development. A single Russian sturgeon mNT exhibited a visible injury throughout its development, probably due to microinjection (Fig. 4; Supplementary Movie 1).

### Use of molecular markers for mNTs identification

To evaluate the genetic origin of the mNTs, molecular analysis of Russian sturgeon and beluga mNTs was performed on the gastrula. The albino sterlet mNTs did not develop sufficiently for identification based on phenotype. All three beluga mNTs presented amplification of the sterlet 247 bp band using the *247*_*Arp* + *247*_*uni* primer pair, while no amplification of the beluga 153 bp band with primer pair *153*_*HHp* + *153*_*uni* was detected, demonstrating that they contained genome of the sterlet recipient only with no contribution of the donor beluga (Supplementary Fig. 2).

After interspecific mSCNT, the Russian sturgeon mNT-4 and the Russian sturgeon mNT-5 did not show any private allele of Russian sturgeon donor and they originated from recipient genome only (Table 3; Supplementary Fig. 3; Supplementary Table 1). The specimens Russian sturgeon mNT-6, Russian sturgeon mNT-7 and Russian sturgeon mNT-8 contained private alleles of Russian sturgeon donor at all informative loci (Supplementary Table 1). The specimens Russian sturgeon mNT-6 and Russian sturgeon mNT-7 showed concurrent occurrence of the recipient genome as evidenced by the presence of recipient’s private alleles in the specimens’ allele phenotypes and from amplification of 247 bp band by primer pair *247*_*ARp* + *247*_*uni* (Table 3). On the contrary, the specimen Russian sturgeon mNT-8 did not possess any recipient private allele and displayed no amplification by primer pair *247*_*ARp* + *247*_*uni* (Supplementary Fig. 3). It showed that this specimen contained only donor’s genome with no contribution from the recipient (Table 3; Supplementary Fig. 3; Supplementary Table 1).

**Table 3:** Molecular analysis using microsatellites markers after interspecific mSCNT.

Specimens after interspecific mSCNT	Marker																		
	*247*_*AR*	*AciG*_*35*		*Afu*_*19*		*Afu*_*68*		*AfuG*_*54*		*AfuG*_*135*		*Aox*_*27*		*Aox*_*45*		*Spl*_*101*		*Spl*_*163*	
		D	R	D	R	D	R	D	R	D	R	D	R	D	R	D	R	D	R
mNT-4	+	−	+	−	+	−	+	−	+	N/A	N/A	−	+	−	+	N/A	N/A	−	+
mNT-5	+	−	+	−	+	−	+	−	+	−	+	−	+	−	+	N/A	N/A	−	+
mNT-6*	+	+	+	+	N/A	+	+	+	N/A	+	+	+	N/A	+	+	+	N/A	+	N/A
mNT-7*	+	+	+	+	N/A	+	+	+	+	+	+	+	+	+	+	+	N/A	+	N/A
mNT-8*	−	+	−	+	N/A	+	N/A	+	N/A	+	N/A	+	N/A	+	−	+	N/A	+	N/A

The Russian sturgeon mNTs-6 and 7 contain alleles from both Russian sturgeon donor and sterlet recipient. The Russian sturgeon mNT-8 dispays only Russian sturgeon donor genome. Name of specimens is from mNT-4 to 8 as they are different from specimen mNT-1 to 3 in Fig. 2.

D = donor; R = recipient; + = informative allele(s) present in allele phenotype of the sample; − = informative allele(s) not present in allele phenotype of the sample; N/A = not informative locus.

*Specimens possessing all informative alleles from the donor.

## Discussion

The main importance of our study lies in the optimization of the animal cloning technique in sturgeon. A major feature of interspecific mSCNT was the production of a gastrula stage embryo that contained only Russian sturgeon donor genome. This unique result after sturgeon SCNT is providing evidence for potential regeneration of populations of large animals with high commercial and ecological value by interspecific SCNT.

In our previous study on interspecific single-SCNT, we established several crucial steps specific to sturgeon biology, obtaining 12% early development of the NTs^11^. Although developed in a separate set of experiment that may complicate direct comparison, the current study using interspecific mSCNT produced higher percentage of early developing transplants (×5.5), even though the surviving embryos did not develop as far as in our former study^11^. We believe that mSCNT is a very promising strategy to increase the clone production because with this technique, we were able to obtain for the first time a gastrula stage mNT of only donor’s origin and two gastrula stage mNTs bearing both the recipient and donor genome. Besides, mSCNT is more effective than single-SCNT since we are able to produce twice the amount of nuclear transplants in half the time of SCNT technique.

Our first hypothesis to explain the higher number of developing transplants using mSCNT is that the transplantation of multiple cells increases the likelihood of placement in the recipient egg optimal for development. A six-fold number of injected fin cells yielded 4- and 1.6-fold early developing Russian sturgeon and beluga mNTs, respectively.

A second hypothesis, suggested by *in vivo* fluorescence analysis where we investigated the fate of freshly dissociated fin cells, is that the mSCNT may have an advantage over single-SCNT due to injection of both intact fin cells and those with altered membranes. Alteration of the donor cell plasma membrane could help the injected exposed nucleus to activate easier the recipient egg. In mammalian SCNT, it is common practice to disrupt the plasma membrane of the somatic cell prior to transplantation^25–27^, although in goldfish it has been shown that injection of the entire fin cell allows embryo development^18^. However, Le Bail *et al*.^18^ reported that the fin cell plasma membrane was spontaneously disrupted seconds after transplantation, something not observed in the present work, *i*.*e*. red stained membranes were visible in 8-cell stage Russian sturgeon mNT (6 hpa). This may indicate that the sterlet ooplasm did not digest the donor plasma membrane. For that reason, we suggest that the hypothesis of the exposed nucleus can be valid in sturgeon cloning. It must be kept in mind that when intact somatic cells were injected, their cytoplasmic content was injected as well. If released from the cells after injection, this interphasic cytoplasm may have interfered with the metaphasic egg cytoplasm and have altered the embryonic clock upon egg activation and the onset of meiosis resumption. We have no clue about the extent of this phenomenon which may explain why some transplants did not develop. However, release of cytoplasm may not be thorough as indicated by the number of cells with their plasma membranes still observed at the 8-cell stage.

After mSCNT, all developing mNTs displayed development similar to the pattern of the sterlet control embryos. Sturgeon eggs may possess a defense mechanism to retain only a single fin cell and to absorb and eliminate other cells. To support that only one fin cell donor activates the recipient egg, we focused on the early development of the mNTs. In sturgeon experiments, both *in vivo*^28^ and *in vitro*^29^, eggs fertilized by multiple spermatozoa resulted in an abnormal number of blastomeres during early development. Iegorova *et al*.^29^ showed that, generally, only one spermatozoon fuses with the egg nucleus and that the sturgeon eggs eliminate the remaining spermatozoa and exhibit a normal phenotype. However, occasionally the surplus spermatozoon/spermatozoa developed further as haploid cells resulting in formation of extra blastomeres^29^. In mSCNT experiments, if we consider fin cells to be analogous to spermatozoa, we can support the hypothesis that only one fin cell activated and triggered the development of the mNTs, because they were characterized by the normal number of blastomeres. However, validation of this hypothesis will require improvement of the mSCNT technique to generate hatched mNTs to assess genotype and ploidy.

We cannot conceal that during development, two Russian sturgeon mNTs exhibited atypically large blastomeres that coexisted with those of normal size. This may have been due to absence of mitotic events at this area or to slower division, either of which could lead to apoptosis, which would explain the development arrest. However, their cleavage division progressed similar to that of the sterlet control group, and the formed blastomeres were normal in number; hence the only difference from the sterlet control group was the large blastomeres from 4-cell stage to late blastula. We cannot support the hypothesis that the size of the Russian sturgeon mNTs’ blastomeres was a factor in the induction of apoptosis, since development was halted at the same stage as the other Russian sturgeon mNTs. In keeping with our hypothesis that a single fin cell activated development, the surface of the larger blastomeres may provide space for the non-expressed donor nucleus digestion by the recipient egg. This can be supported by the fact that, at blastula, the Russian sturgeon mNTs with large blastomeres exhibited no phenotypic difference from the other Russian sturgeon mNTs or those of the sterlet control group. It is interesting to emphasize that the sterlet eggs showed high resistance to stressful conditions. Despite the “rough” manipulation due to microinjection, and the stress of multiple fin cell injection, most mNTs that showed early development reached to the gastrula even when an injury from the microinjection position was visible. This affirms the suggestion of Ginsburg and Dettlaff^28^ of the value of sturgeon species for experimental embryological studies.

Similar to our previous results with single-SCNT^11^, development of both NTs and mNTs from the current study was limited to the gastrula. However, the proportion of blastula mNTs that gastrulated was generally lower than seen with NTs^11^. Thus, after interspecific mSCNT, the number of blastula Russian sturgeon mNTs that reached gastrulation decreased abruptly 17 times compared to 1.4 times in Russian NTs from single-SCNT^11^. Conversely, in albino sterlet mSCNT, the number of blastula albino sterlet mNTs that reached gastrulation was similar to this of albino NTs: 4 and 3.3 times^11^, respectively. The number of blastula beluga mNTs that gastrulated decreased 8.4 times. Therefore, we can suggest that the critical stage in sturgeon cloning could be to reach the early gastrula, as all developing transplants that showed initial cleavage fulfilled the blastula. In actinopterygian SCNT, the critical stage for the NTs to overcome is the mid-blastula^16–18,30^. This unique characteristic within the actinopterygians may be attributed to embryonic genome activation and mitotic checkpoint triggering that take place in the late blastula as has been observed in Russian sturgeon^31^ rather than in the mid-blastula as seen in the higher teleosts. It is reasonable to assume that this applies to all sturgeons. In amphibian SCNT, the critical stage seems to be the late blastula^32,33^, as demonstrated by Dettlaff *et al*.^31^ who noted the similarity in egg structure and cleavage pattern of acipenserids and amphibians. This similarity is also expressed in the process of gastrulation, the changes in the morphology of the embryo, morphogenetic movements, and the fate map of acipenserids being similar with those of anurans^34^. Therefore, SCNT might be more easily employed in sturgeon than in higher teleosts.

After the interspecific mSCNT, 40% of the gastrula stage Russian sturgeon mNTs contained both recipient and donor genome, the first donor DNA integration reported in the embryo with sturgeon SCNT. This can be explained by the use of non-enucleated eggs. In medaka^35–38^ and in zebrafish cloning^39^, after single blastula nuclei transplant into non-enucleated unfertilized eggs, the NTs that grew to the adult stage expressed genetic markers of both donor and recipient. Following Fatira *et al*.^11^, we utilized non-enucleated and non-activated sterlet eggs, which, in teleost SCNT, has been shown to have a positive result in goldfish^18^. In our study, one of the five-gastrula stage Russian sturgeon mNTs possessed only Russian sturgeon donor alleles. Bubenshchikova *et al*.^40^ were the first to hypothesize a yet to be determined mechanism responsible for excluding the recipient nucleus from the NTs. This is the first report of a Russian sturgeon, a critically endangered species, generated from interspecific SCNT. This exclusively donor-DNA-derived embryo is evidence of the potential of assisted reproduction technology to conserve threatened population by interspecific SCNT^25,41,42^. The limitation of our result is that the Russian sturgeon mNT ceased development in the embryonic phase. As already discussed in teleost^43^, fin cells bear a specific differentiated profile based on epigenetic marks which may not be accurately reprogrammed during SCNT. It was shown for example that numerous genes are differentially expressed between embryos obtained after fertilization and after nuclear transfer^44^. It is likely that in the present experiment, none of the donor cells were efficiently reprogrammed. It cannot be excluded either that the somatic cytoplasm incorporated during mSCNT impaired epigenetic reprogramming of the injected nuclei, and that a preliminary reprogramming treatment as in Chenais *et al*.^43^ will be required with our technique.

This Russian sturgeon mNT from donor origin only lost the recipient DNA. It has been recently proposed in teleost^45^ that after SCNT, the egg DNA remains under the first cleavage grove and is scattered without replication in the blastomeres upon successive cleavage, or that it is extruded as a whole with the second polar body, because of an alteration of the meiotic furrow.

Two of the five Russian sturgeon mNTs and the three beluga mNTs displayed only the recipient sterlet genome. This is in accordance with our previous experiment using interspecific single SCNT, in which the obtained NT larva displayed only recipient sterlet genome as a result of unusual disruption in early embryogenesis^11^. Gynogenesis has been observed after fish SCNT when non-enucleated recipient eggs were used: zebrafish intraspecific embryonic cell nuclear transfer resulted in adult diploid NTs (1.6%) that originated only from the recipient^39^. In medaka SCNT, adult NTs (1.0%) exhibited only recipient-derived markers^40^. These authors proposed that the combination of successful diploidization of the recipient nuclei in medaka with no active mitosis in donor-derived nuclei resulted in the formation of parthenogenic individuals^40^. In our experiments we did not diploidized the sterlet recipient genome prior to transplantation, but we know that this already happened accidently in Fatira *et al*.^11^. It cannot be excluded that some alteration of the egg plasma membrane during SCNT prevented the correct extrusion of the second meiotic polar body, resulting in the maintenance of a diploid recipient genome. We strongly suggest that beluga mSCNT will show positive results, as acipenserids can hybridize, resulting in viable progeny^46,47^. Species that hybridize naturally are more likely to perform better in interspecific SCNT^48^, since viable hybrid offspring indicates that nuclear-cytoplasmic compatibility exists between the two species^49^.

The present study provides evidence that interspecific cloning can be used for reproduction of critically endangered large animals. Improvements of the method according to species biology are necessary to develop an efficient tool for conservation of wild populations.

## Methods

### Ethics

The transplantation experiments took place at the Faculty of Fisheries and Protection of Waters, Research Institute of Fish Culture and Hydrobiology, University of South Bohemia in České Budějovice, Czech Republic. All procedures were performed in accordance with national (reference number: 2293/2015-MZE-17214) and institutional guidelines on animal experimentation and care and approved by the Animal Research Committee of the University of South Bohemia in České Budějovice.

### Fish and gamete collection

In mSCNT experiments a single non-sexually-mature 2–3 year old albino sterlet, two Russian sturgeon (3–4 year old), and six beluga (3–4 month old) were used as donor species, and five mature 5–6 year old sterlet females were used as recipient species. *In vitro* fertilization of a sterlet control group was conducted using sperm from five mature male sterlet (6–7 year old). In deep single-SCNT experiments a non-sexually-mature 2–3 year old albino sterlet, and a Russian sturgeon (1–2 year old) were used as donor species, and one mature 6–7 year old sterlet female was used as recipient species. To perform *in vitro* fertilization of the sterlet control group sperm collected from a mature 7–8 year old sterlet male. Ovulation and spermiation were hormonally induced, and eggs and sperm were collected as described by Fatira *et al*.^11^.

### Donor fin cell preparation

In deep single-SCNT experiments caudal fin tissue (~0.5 cm^2^) from albino sterlet (single experiment) and Russian sturgeon (single experiment) was clipped using sterile scissors. In mSCNT experiments caudal fin tissue (~0.5 cm^2^) from albino sterlet (single experiment) and Russian sturgeon (two experiments, each experiment reflects the different cell quantity injected) as well as fin tissue from beluga (two experiments, each experiment reflects the different cell quantity injected) was clipped using sterile scissors. The fin fragments were dissociated into single cells according to Fatira *et al*.^11^ 2–3 h before the mSCNT experiment. In mSCNT experiments the pellet containing fin cells from albino sterlet was suspended in 25 µl phosphate buffered saline (PBS), while the pellets containing Russian sturgeon or beluga fin cells were suspended in either 25 µl or 150 µl PBS. Cell viability and concentration were assessed using a hemocytometer (Bright-Line^TM^ Hemacytometer; Hausser Scientific) after Trypan blue staining (SIGMA-ALDRICH®). The cells were kept at 4 °C until use.

### Nuclear transfer

The recipient sterlet eggs were washed three times with Persian sturgeon artificial coelomic fluid (PSACF)^50^ as described by Fatira *et al*.^11^, and placed in a 6 cm^2^ Petri dish filled with PSACF at 15 °C. Transplantation was conducted using a hydraulic injector (Cell-Tram Oil; Eppendorf, Germany) connected to a micromanipulator (MO-152; Narishige, Japan) under a stereomicroscope (Leica M165 FC.) according to Le Bail *et al*.^18^. To test if there is a favorable position in the large egg of sturgeon for donor nucleus, we performed deep single-SCNT, in which a single donor cell was transplanted into recipient oocytes at the depth of about 1 mm from the animal pole. In deep single-SCNT experiments we followed the transplantation procedure as described in Fatira *et al*.^11^. In mSCNT experiments each egg was held with a 0.7–0.8 mm glass capillary holder so that the animal pole faced the pulled-glass microcapillary needle (inner diameter: 25–28 μm) filled with 5 μl of fin cell suspension. A small quantity of fin cell suspension (~0.2 μl) was transferred into each sterlet egg (for albino sterlet ~329 ± 2 cells/egg, n = 18, for Russian sturgeon ~330 ± 12, n = 128 or ~55 ± 3 cells/egg, n = 34 and for beluga ~240 ± 18, n = 130 or ~40 ± 2 cells/egg, n = 56). After all mSCNT experiments (5 experiments), the albino sterlet mNTs (n = 18), the Russian sturgeon mNTs (n = 162) and the beluga mNTs (n = 186) were incubated in the PSACF (15 °C) for 30–40 min and then activated with filtrated water. Simultaneously, fertilization of the control group with freshly stripped sterlet sperm (5 experiments for mSCNT with 90–93% fertilization rate and a single experiment for deep single-SCNT with 95.7% fertilization rate) was performed to produce control sterlet embryos (n = 351 for mSCNT, n = 187 for deep single-SCNT). We used sterlet eggs with PSACF only (n = 100) as a negative control, activated in filtered water after 30–40 min incubation.

### Treatment and culture of the embryos

Immediately after activation of all transplants and fertilization of the sterlet control group, all embryos were treated with 0.01% tannic acid (SIGMA-ALDRICH®) alternating with filtrated water for 10 min to remove the egg surface stickiness. Development was observed at the two-cell stage, corresponding to 3–4 hpa. At 10 hpa, forceps were used to remove the outer layers of chorion for better observation of the development. Developing embryos were placed in 0.01% penicillin and 0.01% streptomycin in filtered water at 15 °C for three days. All embryos were held at the ambient photoperiod at water temperature of 15 °C.

### Statistical analysis

We used R software (v. 3.5.1) to compare the number of Russian sturgeon and beluga mNTs generated with injection of two numbers of fin cells. Non-developing mNTs and those developing to blastula and gastrula at each donor cell number were compared using Fisher’s exact test. *P* < 0.05 was considered to be significant.

### *In vivo* fluorescence observation of fin cells fate

Prior to interspecific mSCNT, we labeled freshly dissociated fin cells from a Russian sturgeon with Hoechst 33342 (SIGMA-ALDRICH®) and PKH26 Red Fluorescent Cell Linker Kit for General Cell Membrane Labeling (SIGMA-ALDRICH®) according to the manufacturer’s instructions. After washing the stained cells to prevent injection of the dyes, we injected cells into sterlet recipient eggs (n = 2). We monitored the developing embryos and, at the 8 cell-stage we smashed the developing Russian mNTs with a coverslip. The released nuclei were identified with Hoechst 33342, and donor cell plasma membrane was detected with the PKH26. Stained cells were observed under fluorescence microscopy (Leica M165 FC) with ultraviolet light and Red Fluorescent Protein (DsRED) filter linked to the illuminator (Leica Kubler CODIX) to enhance the fluorescence. The merged images were processed with ImageJ software v.1.47.

### Histology

Sturgeon embryos were embedded in plastic to maintain their lipoid-tissue structure. Three Russian mNTs and three sterlet controls at early blastula were placed in Bouin’s fixative for 24 h and subsequently stored in 80% ethanol (EtOH), gradually replaced by 100% EtOH. Thereafter, the embryos were infiltrated with Technovit 7100 in a shaker as follows: 25% Technovit 7100 in EtOH for 12 h, 50% Technovit 7100 in EtOH for 12 h, 75% Technovit 7100 in EtOH for 12 h, 100% Technovit for 24 h (last step repeated twice). After the addition of the Technovit 7100 Hardener I and II, the embryos were placed in a mold for 24 h at −30 °C and subsequently polymerized in an incubator for 24 h at 60 °C. Samples were cut into 4 µm sections using Leica RM2235 and stained with hematoxylin and eosin. Observation of nucleus (purple dot) inside a single blastomere (pink surface) in Russian sturgeon mNTs showed that the formation of blastomeres is the result of development and not due to a stress-response caused by microinjection.

### Molecular genotyping

Genomic DNA was extracted from caudal fin tissue of two Russian sturgeons fin donors, two sterlet egg recipients, one 3–4-month-old beluga (not fin donor) along with five Russian mNTs and three beluga mNTs at gastrula stage using GenElute Mammalian Genomic DNA Miniprep Kit (SIGMA-ALDRICH®) according to the manufacturer’s instructions. The presence of the donor genome in the three beluga mNT embryos was investigated using beluga specific primer pair *153*_*HHp* + *153*_*uni*^24^ that amplifies 153 bp fragment of beluga DNA. Presence of the sterlet recipient genome in beluga mNTs was tested by sterlet specific primer pair *247*_*ARp* + *247*_*uni*^24^, which amplifies 247 bp fragment from sterlet DNA. All reactions were performed according to Havelka *et al*.^24^ in two independent replicates. Because no nuclear DNA marker for identification of the Russian sturgeon genome is available, the presence of donor genome in the five Russian sturgeon mNTs was estimated by parentage-like assignment using nine microsatellite markers: *AciG*_*35*^51^, *Afu*_*19*, *Afu*_*68*^52^, *AfuG*_*54*, *AfuG*_*135*^53^, *Aox*_*27*, *Aox*_*45*^54^, *Spl*_*101*, *and Spl*_*163*^55^. Amplification and microsatellite fragment analysis were carried out according to the protocol described by Havelka *et al*.^56^. Genotypes were scored in GENEIOUS 8.1.9, using Microsatellite Plugin 1.4.4. The complexity of the duplicated sturgeon genome and the state of current microsatellite genotyping make it impossible to reliably determine allele dosage behind a specific peak. Hence, peak pattern was treated as dominant data and interpreted as allele phenotype^57^. Alleles that the Russian sturgeon donor did not share with the sterlet recipient (private alleles) were identified and tracked in allele phenotypes of Russian sturgeon mNTs. Together with microsatellite genotyping, sterlet specific primer pair *247*_*ARp* + *247*_*AR* was used to confirm presence of the sterlet recipient genome in Russian sturgeon mNTs as described above.

## Supplementary information

Supplementary Information

Supplementary Video 1

## References

[1] Ludwig A, Makowiecki D, Benecke N (2009). Further evidence of trans-Atlantic colonization of Western Europe by American Atlantic sturgeons. Archaeofauna.

[2] Birstein VJ, Bemis WE, Waldman JR (1997). The threatened status of acipenseriform species: a summary. Environ. Biol. Fishes.

[3] IUCN. *The IUCN Red List of Threatened Species*. *Version 2018-1*, http://www.iucnredlist.org (Downloaded on 05 July 2018).

[4] Wildt DE (1992). Genetic resource banks for conserving wildlife species: justification, examples and becoming organized on a global basis. Anim. Reprod. Sci..

[5] Lanza RP (2000). Cloning of an endangered species (*Bos gaurus*) using interspecies nuclear transfer. Cloning.

[6] Solti L, Crichton EG, Loskutoff NM, Cseh S (2000). Economical and ecological importance of indigenous livestock and the application of assisted reproduction to their preservation. Theriogenology.

[7] Li Y (2006). Cloned endangered species takin (*Budorcas taxicolor*) by inter-species nuclear transfer and comparison of the blastocyst development with yak (*Bos grunniens*) and bovine. Mol. Reprod. Dev..

[8] Oh HJ (2008). Cloning endangered gray wolves (*Canis lupus*) from somatic cells collected postmortem. Theriogenology.

[9] Gómez MC (2009). Cloning endangered felids using heterospecific donor oocytes and interspecies embryo transfer. Reprod. Fertil. Dev..

[10] Labbé, C., Robles, V. & Herraez, M. P. Cryopreservation of gametes for aquaculture and alternative cell sources for genome preservation. 76–116. In: Advances in aquaculture hatchery technology (Elsevier, 2013).

[11] Fatira E (2018). Application of interspecific Somatic Cell Nuclear Transfer (iSCNT) in sturgeons and an unexpectedly produced gynogenetic sterlet with homozygous quadruple haploid. Sci. Rep..

[12] Lee KY, Huang H, Ju B, Yang Z, Lin S (2002). Cloned zebrafish by nuclear transfer from long-term-cultured cells. Nat. Biotechnol..

[13] Ju B (2003). Development and gene expression of nuclear transplants generated by transplantation of cultured cells nuclei into non-enucleated eggs in the medaka *Oryzias latipes*. Develop. Growth Differ..

[14] Kaftanovskaya E, Motosugi N, Kinoshita M, Ozato K, Wakamatsu Y (2007). Ploidy mosaicism in well-developed nuclear transplants produced by transfer of adult somatic cell nuclei to nonenucleated eggs of medaka (*Oryzias latipes*). Develop. Growth Differ..

[15] Wakamatsu Y (2008). Novel method for the nuclear transfer of adult somatic cells in medaka fish (*Oryzias latipes*): Use of diploidized eggs as recipients. Develop. Growth Differ..

[16] Siripattarapravat K, Pinmee B, Venta PJ, Chang CC, Cibelli JB (2009). Somatic cell nuclear transfer in zebrafish. Nat. Methods.

[17] Luo DJ, Hu W, Chen SP, Zhu ZY (2011). Critical developmental stages for the efficiency of somatic cell nuclear transfer in zebrafish. Int. J. Biol. Sci..

[18] Le Bail PY (2010). Optimization of somatic cell injection in the perspective of nuclear transfer in goldfish. BMC Dev. Biol..

[19] Hochleithner, M. & Gessner, J. The Sturgeons and Paddlefishes (*Acipenseriformes*) of the World – Biology and Aquaculture. 1–248 (AquaTech Publications, 3^rd^ edition, Kitzbühel, 2012).

[20] Gesner, J., Freyhof, J. & Kottelat, M. *Acipenser gueldenstaedtii*. The IUCN Red List of Threatened Species: e.T232A13042340, 10.2305/IUCN.UK.2010-1.RLTS.T232A13042340.en (2010a).

[21] Gesner, J., Chebanov, M. & Freyhof, J. *Huso huso*. The IUCN Red List of Threatened Species 2010: e.T10269A3187455, 10.2305/IUCN.UK.2010-1.RLTS.T10269A3187455.en (2010b).

[22] Akimenko MA, Marí-Beffa M, Becerra J, Géraudie J (2003). Old questions, new tools, and some answers to the mystery of fin regeneration. Dev. Dynam..

[23] Gesner, J., Freyhof, J. & Kottelat, M. *Acipenser ruthenus*. The IUCN Red List of Threatened Species 2010: e.T227A13039007, 10.2305/IUCN.UK.2010-1.RLTS.T227A13039007.en (2010c).

[24] Havelka M, Fujimoto T, Hagihara S, Adachi S, Arai K (2017). Nuclear DNA markers for identification of Beluga and Sterlet sturgeons and their interspecific Bester hybrid. Sci. Rep..

[25] Wilmut I, Schnieke AE, McWhir J, Kind AJ, Campbell KHS (1997). Viable offspring derived from fetal and adult mammalian cells. Nature.

[26] Chesne P (2002). Cloned rabbits produced by nuclear transfer from adult somatic cells. Nat. Biotechnol..

[27] Wani NA, Vettical BS, Hong SB (2017). First cloned Bactrian camel (*Camelus bactrianus*) calf produced by interspecies somatic cell nuclear transfer: A step towards preserving the critically endangered wild Bactrian camels. PLoS One.

[28] Ginsburg, A. S. & Dettlaff, T. A. The Russian Sturgeon *Acipenser Güldenstädti*. Part I. Gametes And Early Development Up To Time Of Hatching. In: Dettlaff, T. A. & Vassetzky, S. G. (eds) Animal Species for Developmental Studies (Springer, Boston, MA, 1991).

[29] Iegorova, V., Pšenička, M., Lebeda, I., Rodina, M. & Saito, T. Polyspermy produces viable haploid/diploid mosaics in sturgeon. *Biol*. *Reprod*. ioy092 (2018). 10.1093/biolre/ioy092 PMC6203876 29701759

[30] Siripattarapravat K (2010). The influence of donor nucleus source on the outcome of zebrafish somatic cell nuclear transfer. Int. J. Dev. Biol..

[31] Dettlaff, T. A., Ginsburg, A. S. & Schmalhausen, O. I. Sturgeon Fishes. Developmental Biology and Aquaculture, ISBN: 3-540-54744-4 (Springer-Verlag Berlin Heidelberg New York, 1993).

[32] Gurdon JB (1962). The developmental capacity of nuclei taken from intestinal epithelium cells of feeding tadpoles. J. Embryol. Exp. Morphol..

[33] Gurdon JB, Laskey RA, Reeves OR (1975). The developmental capacity of nuclei transplanted from keratinized skin cells of adult frogs. J. Embryol. Exp. Morphol..

[34] Ballard WW (1981). Morphogenetic movements and fate maps of vertebrates. Am. Zool..

[35] Niwa K, Ladygina T, Kinoshita M, Ozato K, Wakamatsu Y (1999). Transplantation of blastula nuclei to non-enucleated eggs in the medaka, *Oryzias latipes*. Dev. Growth. Dif..

[36] Niwa K, Kani S, Kinoshita M, Ozato K, Wakamatsu Y (2000). Expression of GFP in nuclear transplants generated by transplantation of embryonic cell nuclei from GFP-transgenic fish into nonenucleated eggs of medaka, *Oryzias latipes*. Cloning.

[37] Wakamatsu, Y. & Ozato, K. *Cloning of Fish*. In: Principles of Cloning, 287–299 (2002).

[38] Bubenshchikova E (2005). Generation of fertile and diploid fish, medaka (*Oryzias latipes*), from nuclear transplantation of blastula and four-somite-stage embryonic cells into nonenucleated unfertilized eggs. *Clon*. Stem Cells.

[39] Hattori M, Hashimoto H, Bubenshchikova E, Wakamatsu Y (2011). Nuclear transfer of embryonic cell nuclei to non-enucleated eggs in zebrafish, *Danio rerio*. Int. J. Biol. Sci..

[40] Bubenshchikova E (2008). Nuclear transplants from adult somatic cells generated by a novel method using diploidized eggs as recipients in medaka fish (*Oryzias latipes*). Clon. Stem Cells.

[41] Kishigami S, Wakayama S, Hosoi Y, Iritani A, Wakayama T (2008). Somatic cell nuclear transfer: Infinite reproduction of a unique diploid genome. Exp. Cell Res..

[42] Loi P, Iuso D, Czernik M, Zacchini F, Ptak G (2013). Towards storage of cells and gametes in dry form. Trends Biotechnol..

[43] Chenais N (2019). Nuclear import of Xenopus egg extract components into cultured cells for reprogramming purposes: a case study on goldfish fin cells. Sci. Rep..

[44] Luo D (2009). Identification of differentially expressed genes between cloned and zygote-developing zebrafish (*Danio rerio*) embryos at the dome stage using suppression subtractive hybridization. Biol. Reprod..

[45] Rouillon, C., Depincé, A., Chênais, N., Le Bail, P. Y. & Labbé, C. Somatic cell nuclear transfer in non-enucleated goldfish oocytes: understanding DNA fate during meiosis resumption and first cellular division. *Sci*. *Rep*. (submitted), bioRxiv 630194; doi: 10.1101/630194. 10.1038/s41598-019-48096-2 PMC6713701 31462687

[46] Gorshkova G, Gorshkov S, Gordin H, Knibb W (1996). Karyological study in hybrids of beluga, *Huso huso* (L.) and the Russian sturgeon *Acipenser gueldenstaedti* brandt. Israel J. Aquacult..

[47] Havelka M (2014). Fertility of a spontaneous hexaploid male Siberian sturgeon, *Acipenser baerii*. BMC Genet..

[48] Lagutina I (2010). Development, embryonic genome activity and mitochondrial characteristics of bovine-pig inter-family nuclear transfer embryos. Reproduction.

[49] Mastromonaco GF, Favetta LA, Smith LC, Filion F, King WA (2007). The influence of nuclear content on developmental competence of gaur x cattle hybrid *in vitro* fertilized and somatic cell nuclear transfer embryos. Biol. Reprod..

[50] Sohrabnezhad M, Kalbassi MR, Nazari RM, Bahmani M (2006). Short-term storage of Persian sturgeon (*Acipenser persicus*) ova in artificial media and coelomic fluid. J. Appl. Ichthyol..

[51] Börk K (2008). Development of new microsatellite primers for green and white sturgeon. Conserv. Genet..

[52] May B, Krueger CC, Kincaid HL (1997). Genetic variation at microsatellite loci in sturgeon: primer sequence homology in *Acipenser* and *Scaphirhynchus*. Can. J. Fish. Aquat. Sci..

[53] Welsh AB, Blumberg M, May B (2003). Identification of microsatellite loci in lake sturgeon, *Acipenser fulvescens*, and their variability in green sturgeon, *A*. *medirostris*. Mol. Ecol. Notes.

[54] King TL, Lubinski BA, Spidle AP, Microsatellite DNA (2001). variation in Atlantic sturgeon (*Acipenser oxyrinchus oxyrinchus*) and cross-species amplification in the Acipenseridae. Conserv. Genet..

[55] McQuown EC (2000). Microsatellite analysis of genetic variation in Sturgeon: new primer sequences for *Scaphirhynchus* and *Acipenser*. Trans. Am. Fish. Soc..

[56] Havelka M, Hulák M, Bailie DA, Prodöhl PA, Flajšhans M (2013). Extensive genome duplications in sturgeons: new evidence from microsatellite data. J. Appl. Ichthyol..

[57] Rodzen JA, Famula TR, May B (2004). Estimation of parentage and relatedness in the polyploid white sturgeon (*Acipenser transmontanus*) using a dominant marker approach for duplicated microsatellite loci. Aquaculture.

