# Supplementary material for: A newly developed cloning technique in sturgeons; an important step towards recovering endangered species
Source: Sci Rep. 2019 Jul 18;9:10453. doi: 10.1038/s41598-019-46892-4 (PMC6639416; doi:10.1038/s41598-019-46892-4)
Supplement: Supplementary file 1 — Supplementary Information [file 41598_2019_46892_MOESM1_ESM.pdf]

Supplementary Information

A newly developed cloning technique in sturgeons; an important step towards recovering endangered species

Effrosyni Fatira[1,*], Miloš Havelka[2], Catherine Labbé[3], Alexandra Depincé[3], Martin Pšenička[1], Taiju Saito[1,2]

[1]Faculty of Fisheries and Protection of Waters, South Bohemian Research Center of Aquaculture and Biodiversity of Hydrocenoses, University of South Bohemia in České Budějovice, Zátiší 728/II, 389 25 Vodňany, Czech Republic. [2]South Ehime Fisheries Research Center, Ehime University, Ainan, Ehime 798-4206, Japan. [3]INRA, Fish Physiology and Genomics department, Campus de Beaulieu, F-35000 Rennes, France.

*Correspondence and request for materials should be addressed to E.F.

effrosini.fatira@gmail.com

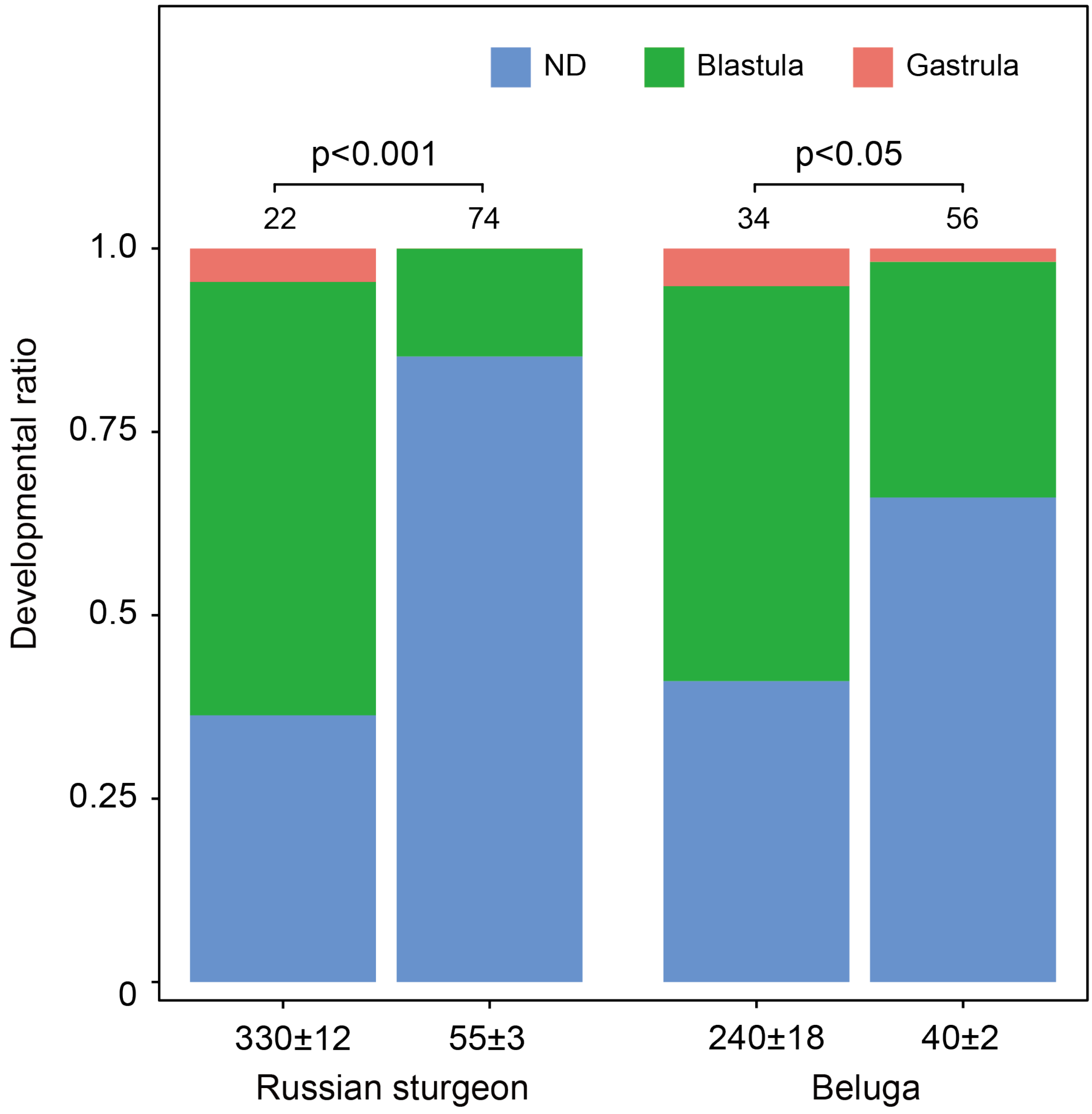

Supplementary Fig. 1. Higher number of embryos were activated and developed further than blastula stage when higher number of donor cells was injected. The number under the bars indicates the number of transplanted donor cells per egg. The numbers above the bars indicate the total number of embryos used for this analysis. ND= non-developing embryo; Blastula= embryos developed until blastula; Gastrula= embryos developed until gastrula.

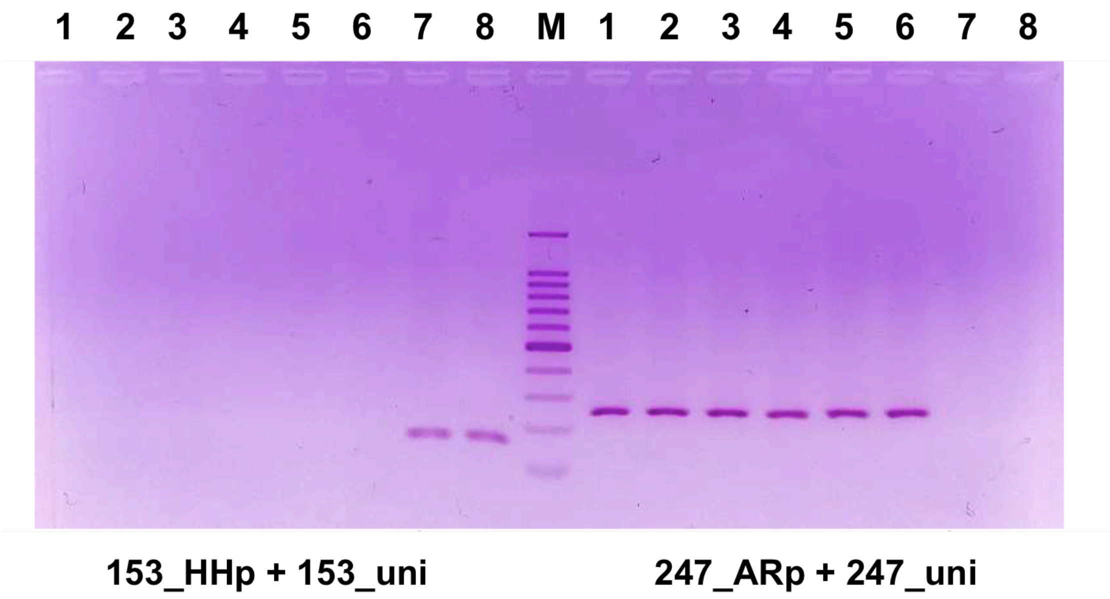

Supplementary Fig. 2. Amplification by beluga specific primer pair *153_HHp* + *153_uni* and sterlet specific primer pair *247_ARp* + *247_uni*.

1-2 = beluga mNT-1; 3-4 = beluga mNT-2; 5-6 = beluga mNT-3; 7-8 beluga (fin-tissue control sample); M = 100 – 1500bp DNA ladder.

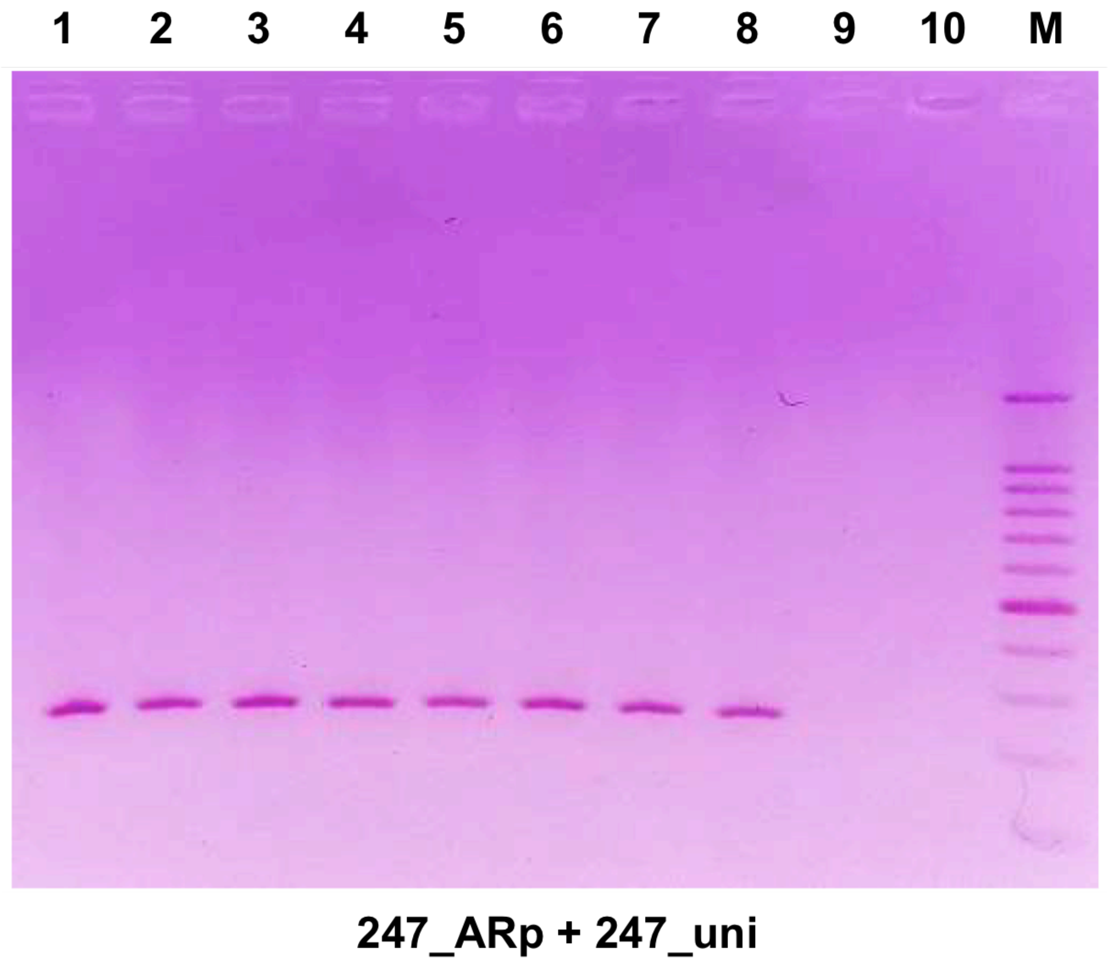

Supplementary Fig. 3. Amplification by sterlet specific primer pair *247_ARp* + *247_uni*. 1-2 = Russian sturgeon mNT-4; 3-4 = Russian sturgeon mNT-5; 5-6 = Russian sturgeon mNT-6; 7-8 = Russian sturgeon mNT-7; 9-10 = Russian sturgeon mNT-8; M = 100 – 1500bp DNA ladder.

Supplementary Movie 1. Development of a Russian sturgeon mNT that exhibit large blastomeres together with the normal size ones and a visible injury due to microinjection. The injury remains throughout the development (gastrula-blastopore formation), demonstrating the resilience of sturgeon embryos.

Supplementary Table 1. Genotyping results of Russian sturgeon multiple fin nuclear transfer specimens (Russian mNT-4 to 8) at 9 microsatellite loci.

AciG_35

ID	Allele 1	Allele 2	Allele 3	Allele 4	Allele 5	Allele 6	Allele 7	Allele 8
Russian Donor_I	268		276	280	284	286	288	292
Sterlet Recipient_I		272			284			292
Russian mNT-4		272						292
Russian mNT-5		272						292

ID	Allele 1	Allele 2	Allele 3	Allele 4	Allele 5	Allele 6	Allele 7	Allele 8	Allele 9
Russian Donor_II	268		276	280	284	286	290	292	296
Sterlet Recipient_II		272			284				
Russian mNT-6	268	272	276	280	284	286	290	292	296
Russian mNT-7	268	272	276	280	284	286	290	292	296
Russian mNT-8	268		276	280	284	286	290	292	296

Afu_19

ID	Allele 1	Allele 2	Allele 3	Allele 4
Russian Donor_I	153	156		165
Sterlet Recipient_I		156	159	
Russian mNT-4		156		
Russian mNT-5			159	

ID	Allele 1	Allele 2	Allele 3	Allele 4
Russian Donor_II	153	156	159	165
Sterlet Recipient_II		156		
Russian mNT-6	153	156	159	165
Russian mNT-7	153	156	159	165
Russian mNT-8	153	156	159	165

Afu_68

ID	Allele 1	Allele 2	Allele 3	Allele 4	Allele 5	Allele 6
Russian Donor_I	150	154	162		210	234
Sterlet Recipient_I				206		
Russian mNT-4				206		
Russian mNT-5				206		

ID	Allele 1	Allele 2	Allele 3	Allele 4	Allele 5	Allele 6	Allele 7
Russian Donor_II	150	154	162		210		266
Sterlet Recipient_II				198		238	
Russian mNT-6	150	154	162	198	210		266
Russian mNT-7	150	154	162		210	238	266
Russian mNT-8	150	154	162		210		266

AfuG_54

ID	Allele 1	Allele 2	Allele 3	Allele 4	Allele 5	Allele 6
Russian Donor_I			262	278	286	294
Sterlet Recipient_I	240	258				
Russian mNT-4	240	258				
Russian mNT-5	240	258				

ID	Allele 1	Allele 2	Allele 3	Allele 4	Allele 5	Allele 6	Allele 7	Allele 8
Russian Donor_II		258		264	278	282	286	294
Sterlet Recipient_II	240		262					294
Russian mNT-6		258		264	278	282	286	294
Russian mNT-7	240	258	262	264	278	282	286	294
Russian mNT-8		258		264	278	282	286	294

AfuG_135				
**ID**	**Allele 1**	**Allele 2**	**Allele 3**	**Allele 4**
Russian Donor_I	204		224	228
Sterlet Recipient_I	204	208		
Russian mNT-4	204			
Russian mNT-5		208		

**ID**	**Allele 1**	**Allele 2**	**Allele 3**	**Allele 4**
Russian Donor_II	198	204		224
Sterlet Recipient_II			208	224
Russian mNT-6	198	204	208	224
Russian mNT-7	198	204	208	224
Russian mNT-8	198	204		224

Aox_27					
**ID**	**Allele 1**	**Allele 2**	**Allele 3**	**Allele 4**	**Allele 5**
Russian Donor_I		152	156	160	164
Sterlet Recipient_I	136				
Russian mNT-4	136				
Russian mNT-5	136				

**ID**	**Allele 1**	**Allele 2**	**Allele 3**	**Allele 4**	**Allele 5**
Russian Donor_II	132		152	160	164
Sterlet Recipient_II	132	136			
Russian mNT-6	132		152	160	164
Russian mNT-7	132	136	152	160	164
Russian mNT-8	132		152	160	164

Aox_45

ID	Allele 1	Allele 2	Allele 3	Allele 4
Russian Donor_I	136	145	161	172
Sterlet Recipient_I		145		
Russian mNT-4		145		
Russian mNT-5		145		

ID	Allele 1	Allele 2	Allele 3	Allele 4	Allele 5
Russian Donor_II	133			157	172
Sterlet Recipient_II		136	139		
Russian mNT-6	133		139	157	172
Russian mNT-7	133	136		157	172
Russian mNT-8	133			157	172

Spl_101

ID	Allele 1	Allele 2	Allele 3
Russian Donor_I	304	316	320
Sterlet Recipient_I		316	
Russian mNT-4		316	
Russian mNT-5		316	

ID	Allele 1	Allele 2	Allele 3	Allele 4
Russian Donor_II	304	316	320	
Sterlet Recipient_II			320	340
Russian mNT-6	304	316	320	
Russian mNT-7	304	316	320	
Russian mNT-8	304	316	320	

Spl_163				
**ID**	**Allele 1**	**Allele 2**	**Allele 3**	**Allele 4**
Russian Donor_I	196	204	220	228
Sterlet Recipient_I			220	
Russian mNT-4			220	
Russian mNT-5			220	

**ID**	**Allele 1**	**Allele 2**	**Allele 3**	**Allele 4**	**Allele 5**
Russian Donor_II	196	204		220	236
Sterlet Recipient_II			208	220	
Russian mNT-6	196	204	208	220	236
Russian mNT-7	196	204		220	236
Russian mNT-8	196	204		220	236

Supplementary Table 2. Intraspecific and interspecific deep single-SCNT and sterlet control group. Deep donor-cell injection into the centre of the recipient egg revealed no initial cleavage.

Experimental group	Total number of eggs	Initial embryonic cleavage (%)
Fertilized sterlet control	187	179 (95.7)
Intraspecific	120	0 (0.0)
Interspecific	130	0 (0.0)
